# One-day dual-tracer examination in neuroendocrine neoplasms: a real advantage of low activity LAFOV PET imaging

**DOI:** 10.1007/s00259-025-07073-w

**Published:** 2025-01-30

**Authors:** Eduardo Calderón, Lena S. Kiefer, Fabian P. Schmidt, Wenhong Lan, Andreas S. Brendlin, Christian P. Reinert, Stephan Singer, Gerald Reischl, Martina Hinterleitner, Helmut Dittmann, Christian la Fougère, Nils F. Trautwein

**Affiliations:** 1 Nuclear Medicine and Clinical Molecular Imaging, University Hospital Tuebingen, Otfried-Mueller-Str. 14, 72076 Tuebingen, Germany; 2 Werner Siemens Imaging Center, Preclinical Imaging and Radiopharmacy, Eberhard-Karls University, Roentgenweg 13, 72076 Tuebingen, Germany; 3 Diagnostic and Interventional Radiology, University Hospital Tuebingen, Hoppe-Seyler-Str. 3, 72076 Tuebingen, Germany; 4 Department of Pathology, University Hospital Tuebingen, Liebermeisterstr. 8, 72076 Tuebingen, Germany; 5 Medical Oncology and Pneumology (Internal Medicine VIII), University Hospital Tuebingen, Otfried- Mueller-Str. 14, 72076 Tuebingen, Germany; 6 University Hospital Tuebingen, ENETS Center of Excellence, Otfried-Mueller-Str. 14, 72076 Tuebingen, Germany; 7 DFG Cluster of Excellence 2180 ‘Image-Guided and Functional Instructed Tumor Therapy’ (iFIT), University of Tuebingen, Roentgenweg 11, 72076 Tuebingen, Germany; 8 German Cancer Consortium (DKTK), German Cancer Research Center (DKFZ) Partner Site Tuebingen, Auf der Morgenstelle 15, 72076 Tuebingen, Germany

**Keywords:** Dual-tracer PET, LAFOV PET/CT, Low activity [^18^F]FDG PET, Neuroendocrine neoplasms

## Abstract

**Purpose:**

Somatostatin receptor (SSTR)-PET is crucial for effective treatment stratification of neuroendocrine neoplasms (NENs). In highly proliferating or poorly differentiated NENs, dual-tracer approaches using additional [^18^F]FDG PET can effectively identify SSTR-negative disease, usually requiring separate imaging sessions. We evaluated the feasibility of a one-day dual-tracer imaging protocol with a low activity [^18^F]FDG PET followed by an SSTR-PET using the recently introduced [^18^F]SiFA*lin*-TATE tracer in a long axial field-of-view (LAFOV) PET/CT scanner and its implications in patient management.

**Methods:**

Twenty NEN patients were included in this study. Initially, a low activity [^18^F]FDG PET was performed (0.5 ± 0.01 MBq/kg; PET scan 60 min p.i.). After 4.2 ± 0.09 h after completion of the [^18^F]FDG PET, a standard activity of [^18^F]SiFA*lin*-TATE was administered (3.0 MBq/kg; PET scan 90 min p.i.). To ensure the quantification accuracy of the second scan, we evaluated the potential impact of residual [^18^F]FDG activity by segmenting organs with minimal physiological SSTR-tracer uptake, such as the brain and myocardium, and assessing the activity concentrations (ACTs) of tumor lesions. Residual tumor lesion ACTs of [^18^F]FDG were calculated by factoring fluorine-18 decay, identifying a maximum residual ACT of 15% (R15%). To account for increased [^18^F]FDG trapping over time, higher residual ACTs of 20% (R20%) were considered. These simulated [^18^F]FDG ACTs were compared with those measured in the second PET scan with [^18^F]SiFA*lin*-TATE. The influence of the dual-tracer PET/CT results on therapeutic strategies was evaluated.

**Results:**

[^18^F]FDG cerebral uptake significantly decreased in the subsequent SSTR-PET (mean uptake [^18^F]FDG: SUV_mean_ 6.0 ± 0.4; mean uptake in [^18^F]SiFA*lin*-TATE PET: SUV_mean_ 0.2 ± 0.01; *p* < 0.0001); with similar results recorded for the myocardium. Simulated residual [^18^F]FDG ACTs represented only a minimal percentage of ACTs measured in the tumor lesions from the second PET scan (R15%: mean 5.2 ± 0.9% and R20%: mean 6.8 ± 1.2%), indicating only minimal residual activity of [^18^F]FDG that might interfere with the second PET scan using [^18^F]SiFA*lin*-TATE and preserved semi-quantification of the latter. Dual-tracer PET/CT findings directly influenced changes in therapy plans in eleven (55%) of the examined patients.

**Conclusion:**

LAFOV PET scanners enable a one-day dual-tracer protocol, providing diagnostic image quality while preserving the semi-quantification of two ^18^F-labeled radiotracers, potentially simplifying the assessment of tumor biology and improving the clinical patient management while reducing logistical challenges. Additionally, low-activity PET imaging facilitates one-day dual-tracer PET examinations.

**Supplementary Information:**

The online version contains supplementary material available at 10.1007/s00259-025-07073-w.

## Introduction

Neuroendocrine neoplasms (NENs) comprise a group of heterogeneous tumors originating from various anatomical sites, which are subcategorized into well-differentiated neuroendocrine tumors (NETs) and poorly differentiated neuroendocrine carcinomas (NECs) [1, 2]. Moreover, NETs are subdivided according to their proliferation index in G1 (Ki67 < 3%), G2 (Ki67 3–20%), and G3 (Ki67 > 20%). Well-differentiated NETs are usually characterized by the overexpression of somatostatin receptors (SSTRs) [3], providing a viable target structure for theranostic approaches [4]. In this context, PET imaging is performed through a non-invasive assessment of SSTR expression using radiolabeled somatostatin analogues (SSAs) [5], where [^68^Ga]Ga-DOTA-SSAs are the current gold standard. New ^18^F-labeled radiotracers, such as [^18^F]SiFA*lin*-TATE, have recently been established in clinical practice [6] and might facilitate access to SSTR-PET imaging due to the longer half-life of fluorine-18 and the associated higher production scalability.

Regarding clinical management of patients with NENs, SSTR-PET imaging is crucial for the assessment of disease extent and prognosis, and specifically for adequate patient selection for peptide receptor radionuclide therapy (PRRT) with [^177^Lu]Lu-DOTA-TATE, which has proven to be an essential therapy option especially considered for G1/G2 gastroenteropancreatic NETs [7–9]. Besides this patient group, the NETTER2 trial recently revealed promising results for [^177^Lu]Lu-DOTA-TATE PRRT as a first-line therapy in high-proliferating G2 (≥ 10%) and even G3 (< 55%) tumors [10]. However, NENs with a Ki67 greater than 10% or poor differentiation may exhibit tumor biology changes, leading to increased glucose metabolism and decreased SSTR expression [11]. In these patients, the entire disease burden would be potentially underrepresented in the SSTR-PET, thus leading to inappropriate therapy stratification and worsened outcomes, e.g., for SSTR-targeted therapies.

To address this problem and assist the clinical management of patients with high-grade NENs, dual-tracer PET approaches combining [^18^F]FDG and radiolabeled SSAs have been proposed [12–14]. However, conventional PET/CT scanners with a short axial field-of-view (SAFOV) necessitate two separate imaging sessions on different days to prevent “spill-over” between the applied radiotracers.

New-generation PET/CT scanners with a long axial field-of-view (LAFOV) offer technical advantages compared to SAFOV PET/CT scanners due to their significantly higher sensitivity up to a factor of 10 [15, 16], leading to many new possibilities in hybrid imaging, including the activity reduction of radiotracers. Thereby, activity reductions down to 2.0 MBq/kg of [^18^F]FDG have been proposed and introduced in our standard clinical practice [17]. Furthermore, a previous study showed that further activity reductions down to 0.5 MBq/kg [^18^F]FDG with a 10 min PET acquisition time offers reliable diagnostic performance with preserved lesion detection rate and accurate quantification compared to standard activity (3.0 MBq/kg) PET images [18]. This relevant activity reduction can shorten the required interval between two separate PET scans, making quantifiable dual-tracer PET examinations feasible in one day.

Therefore, we decided to explore the feasibility of a one-day dual-tracer PET imaging protocol for patients with NENs (Ki67 ≥ 10%), combining a low activity [^18^F]FDG PET with 0.5 MBq/kg followed by a standard activity [^18^F]SiFA*lin*-TATE PET with 3.0 MBq/kg and to evaluate its impact in the clinical management of NEN patients.

## Materials and methods

### Study cohort

This study was based on a prospective PET/CT registry, including 20 patients with histologically confirmed G2 (≥ 10%) and G3 (> 20%) NEN. The [^18^F]FDG PET and SSTR-PET with [^18^F]SiFAlin-TATE were performed on a LAFOV PET/CT scanner (Siemens Biograph Vision Quadra, Siemens Healthineers, Knoxville, TN, USA). Written informed consent was obtained from all patients prior to the PET/CT examination. The study was approved by the Institutional Review Board of the University Hospital of Tuebingen (#082/2024BO2).

### Dual-Tracer PET protocol and PET image reconstruction

Detailed information on applied activity and elapsed time between PET scans are provided in Supplementary Table 1. Patients first received a low activity [^18^F]FDG PET after an intravenous (i.v.) injection of 0.5 ± 0.01 MBq/kg. A 10 min PET image acquisition was started at 60 min post-injection (p.i.). Whole-body scans were acquired in supine position and a single bed position (106 cm) covering an area from the skull apex to the mid-thighs. Patients were required to fast for at least 10 h prior to the examination. A venous blood glucose measurement was conducted to confirm blood glucose levels ≤ 200 mg/dl, as described by the European Association of Nuclear Medicine (EANM) procedures guidelines [19]. A low-dose CT scan for attenuation correction was performed before PET acquisition.

A minimum of three and a half hours (mean 4.2 ± 0.09 h) after completing the [^18^F]FDG PET was kept until i.v. injection of 3.0 MBq/kg [^18^F]SiFA*lin*-TATE. Afterward, a 5 min PET image acquisition was started at 90 min p.i., as previously described [20]. [^18^F]SiFA*lin*-TATE was synthesized following a modified procedure of Lindner et al. [21]. A full diagnostic contrast-enhanced dual-energy CT scan in arterial and portal venous phase was acquired and used for attenuation correction. Technical specificities of CT scan protocols are provided in Supplementary Table 2. The average time span until completion of both PET/CT examinations was 6.7 ± 0.09 h. Patients were allowed to leave our center after completion of the first scan and return later for the second PET examination.

PET reconstruction was performed according to the standard clinical reconstruction protocol, with an Ordinary-Poisson Ordered-Subsets Expectation-Maximization algorithm with four iterations and five subsets (OP-OSEM 4i5s), using point-spread-function (PSF) modeling, time-of-flight (TOF) information. For low activity [^18^F]FDG scans, images were reconstructed in both the high-sensitivity mode (HS) and ultra-high sensitivity (UHS) mode with an acceptance angle of 18° and 52°, respectively. For two patients, reconstructions in UHS mode were not available. Images were reconstructed with a 440 × 440 × 645 matrix and a 1.65 × 1.65 × 1.65 mm^3^ isotropic voxel size. No image filter was applied.

### PET image and residual [^18^F]FDG activity analysis

Two experienced nuclear medicine physicians with at least three years of experience in hybrid imaging performed image analysis in consensus reading, analyzing both PET scans simultaneously, thereby reflecting the procedure in clinical practice. Affinity Hybrid Viewer software (Version 3.0.5, Hermes Medical Solution, Sweden) was used for image analysis. For blood pool measurements, the SUV_mean_ of a 3 cm^3^ cylindrical VOI placed in the thoracic descending aorta was recorded in both PET images. Furthermore, a 14 m^3^ VOI was used to measure background uptake in the healthy tissue of the liver lobe.

To evaluate the residual [^18^F]FDG activity in the subsequent [^18^F]SiFA*lin*-TATE PET scan, the brain parenchyma and the myocardium, which are known to have physiological high glucose metabolism but no physiological SSTR tracer uptake, were segmented and assessed. Thereby, a whole-brain threshold-based automatic segmentation was performed, choosing a threshold of 2 x SUV_mean_ of the blood pool volume of interest (VOI). In the case of the myocardium, a 1 cm^3^ cylindrical VOI was placed in the posterior myocardial wall of the left ventricle. Segmented VOIs were consecutively co-registered to the corresponding [^18^F]SiFA*lin*-TATE PET/CT examination, and the magnitude of SUV_mean_ was assessed for residual [^18^F]FDG activity.

Furthermore, to assess the potential impact of residual [^18^F]FDG activity on [^18^F]SiFA*lin*-TATE semi-quantification, we first determined the activity concentrations (ACTs) of all segmented tumor lesions from the [^18^F]FDG PET scan. Secondly, we calculated the residual [^18^F]FDG ACTs present when performing the second PET scan considering the decay of fluorine-18 (half-life 109.8 min). The estimated mean residual ACTs were 12% of the initially measured ACTs in the [^18^F]FDG PET, considering the mean elapsed half-lives of 3.1 ± 0.05 between examinations. In this study, the shortest time interval between the scans was 298 min (approx. 5 h), corresponding to a maximum residual ACT of 15% (R15%).

To account for this worst-case scenario, we used the estimated R15% ACTs for each tumor lesion and determined the percentual proportion in relation to tumor lesion ACTs obtained from the [^18^F]SiFA*lin*-TATE PET scan. However, it is recognized that in certain malignancies, the uptake of [^18^F]FDG can further increase over time. This phenomenon remains to be studied for NENs. However, to address this issue and to simulate an increase in [^18^F]FDG uptake over time, we incorporated a higher estimated residual [^18^F]FDG activity of 20% (R20%) in our analysis.

The coefficient of variation (CoV) was calculated to assess objective image noise on [^18^F]FDG images, as previously described [22]. The recommended CoV of < 15% by the EANM and the European Federation of Organisations for Medical Physics (EFOMP) was considered for interpretation of adequate noise performance [23].

### Tumor lesion segmentation, semi-quantification, and assessment of metabolic tumor volume

A maximum of five representative lesions (lesion volume ≥ 0.5 mL) per organ for each patient were segmented. Tumor lesion uptake was quantified by measuring the SUV_mean_, SUV_peak_, and SUV_SD_ in a VOI using a 41% threshold. VOIs were manually segmented where pathological uptake suspicious for malignancy was recorded. The tumor-to-liver ratio (TLR) was calculated as previously described [21]. Lesions with a TLR equal to or below 1.0 were then categorized as FDG or SSTR negative. Considering the dual-tracer uptake, lesions were classified as follows: FDG+/SSTR-, FDG+/SSTR+, and FDG-/SSTR+. The metabolic tumor volume of [^18^F]FDG (FDG-MTV) images was segmented using a threshold of SUV 4, as described previously [24]. For the SSTR-PET, the molecular tumor volumes (SSTR-MTV) were assessed using a threshold of 1.5 times the SUV_mean_ plus 2 times the standard deviation (SD) of the liver VOI [25].

$$SSTR-MTV = SU{V_{tumor}}\> > 1.5 \times SUVmea{n_{liver}} + 2 \times S{D_{liver}}$$

### Impact on clinical management

To assess the impact of dual-tracer PET on the clinical management of patients, information on the current stage of disease and treatment prior to dual-tracer PET was collected. In addition, we examined the decision-making of the interdisciplinary tumor board (ITB) involving dual-tracer PET findings. In particular, PET parameters that significantly influenced these decisions, i.e., FDG-MTV and SSTR-MTV and individual dual-tracer uptake of tumor lesions, were analyzed. Subsequent treatment changes were documented. The mentioned radiosensitizing (RS) regimen denotes a chemotherapy protocol previously outlined with capecitabine and temozolomide [26], designed to complement and improve the effectiveness of PRRT in high proliferating NENs.

### Statistical analysis

Statistical analysis and figures were performed using GraphPad Prism (Version 9.4.1, GraphPad Software, San Diego, CA, USA) and an open-source software (SankeyMATIC^®^). D’Agostino & Pearson test was performed to test for normality distribution. Continuous variables were compared with the non-parametric Wilcoxon matched-pairs signed rank test and the Kruskal-Wallis test when comparing more than two groups, followed by Dunn´s multiple comparisons. *p* values < 0.05 were considered statistically significant. Summary statistics are presented as mean and standard error (SE).

## Results

### Patient characteristics

Table 1 summarizes the clinical characteristics of the study population. Imaging procedures and time intervals are presented in Supplementary Table 1. Twenty patients were scanned according to our protocol: 12 (60%) males and 8 (40%) females. All patients had a histologically confirmed diagnosis of an NEN with a Ki67 index ≥ 10%.

**Table 1:** Patient characteristics of the study cohort

Patient characteristics	
Age (y)	67.2 ± 9.3
Sex– n (%)	
Male	12 (60%)
Female	8 (40%)
Primary tumor site– n (%)	
Midgut	4 (20%)
Pancreas	5 (25%)
CUP	7 (35%)
Lung	4 (20%)
Grading– n (%)	
G2 (Ki67: 10–20%)	5 (25%)
G3 (Ki67: >20%)	15 (75%)
Previous therapy– n (%)	
Surgery	11 (55%)
SSA	5 (25%)
Chemotherapy	13 (65%)
PRRT	2 (10%)
SIRT	3 (15%)
Everolimus	1 (5%)

CUP = cancer of unknown primary, G = grading, SSA = somatostatin analogue, PRRT = peptide receptor radionuclide therapy, SIRT = selective internal radiation therapy

### Low activity [^18^F]FDG PET and residual [^18^F]FDG activity

**Fig. 1:**
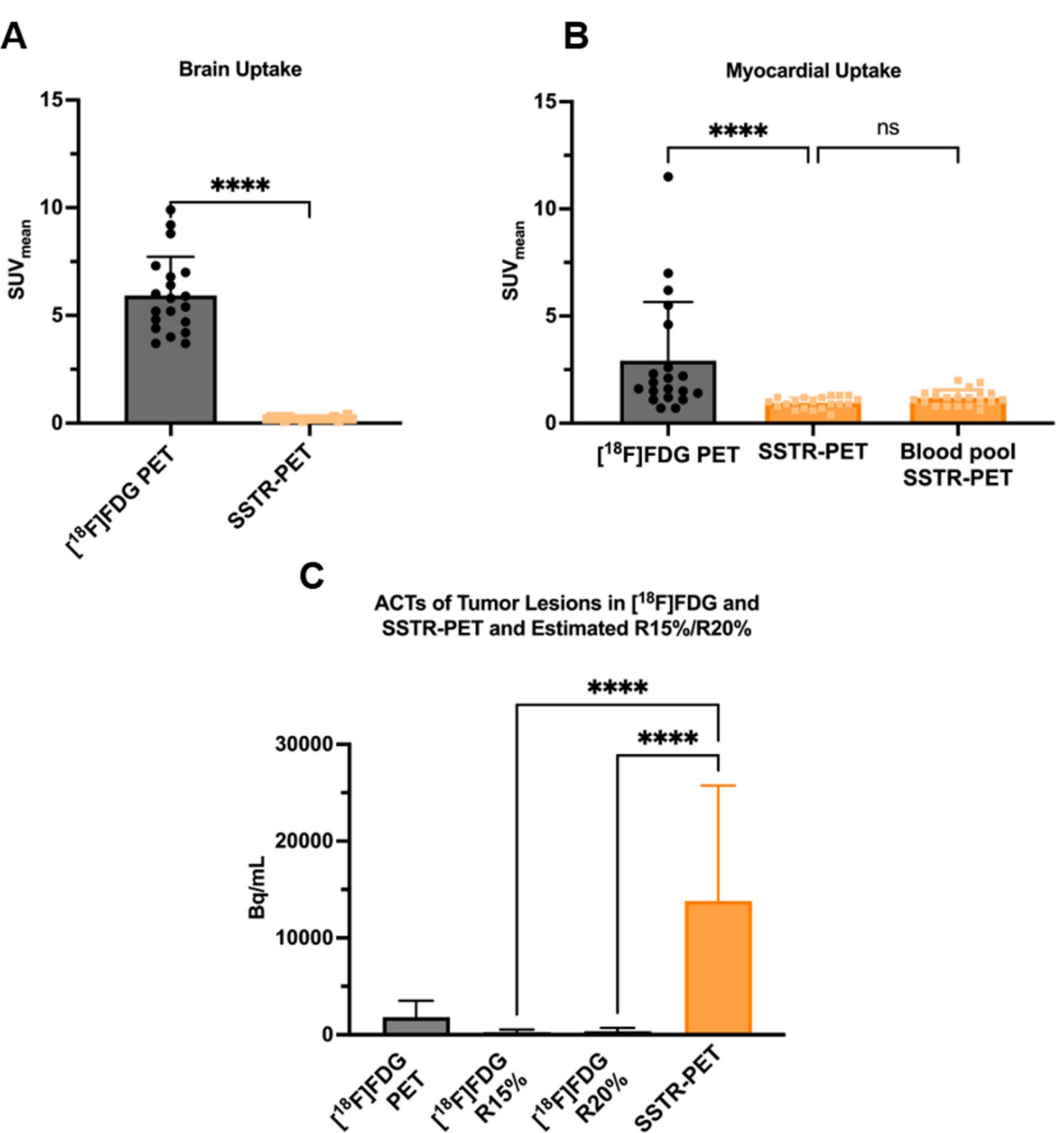
Column bars displaying SUV_mean_ values of brain parenchyma (**A**) and myocardium (**B**) and activity concentrations (ACTs) of tumor lesions (**C**) in the [^18^F]FDG and SSTR-PET. No relevant residual [^18^F]FDG activity was measured in brain parenchyma in subsequent SSTR-PET images. For the myocardium, a similar decrease in uptake values was noted, with assessed uptake values in the myocardium of SSTR-PET images showing no statistically significant difference from blood pool measurements. The estimated residual ACTs of [^18^F]FDG activity (R15%/R20%) are significantly lower in comparison to tumor ACTs observed in the SSTR-PET (*p* < 0.0001). Each bar represents the mean, while the error bars represent the standard deviation. Scatter plots represent individual values. Wilcoxon matched-pairs signed ranked test, and the Kruskal-Wallis test were performed to compare uptake values. ns = non-significant. **** refers to p < 0.001

Semi-quantitative evaluation of brain and myocardial tracer uptake and activity concentrations of segmented tumor lesions, as well as the estimated residual [^18^F]FDG activity concentrations (R15% and R20%), are displayed in Fig. 1. The mean elapsed half-lives of fluorine-18 between both PET data acquisitions were 3.1 ± 0.05. Cerebral [^18^F]FDG uptake was assessed in the first PET images (mean [^18^F]FDG uptake: SUV_mean_ 6.0 ± 0.4) and showed a statistically significant decrease in the corresponding subsequent PET scans (mean uptake in [^18^F]SiFA*lin*-TATE PET: SUV_mean_ 0.2 ± 0.01, *p* < 0.001). Similar results were recorded for the myocardium (mean [^18^F]FDG uptake: SUV_mean_ 2.9 ± 0.6; mean uptake in [^18^F]SiFA*lin*-TATE PET: SUV_mean_ 0.9 ± 0.05, *p* < 0.001). The mean SUV_mean_ values measured in the myocardium from the SSTR-PET scan were not statistically significantly different from those of blood pool measurements (mean SUV_mean_ blood pool 1.1 ± 0.08, *p* = 0.21).

**Fig. 2:**
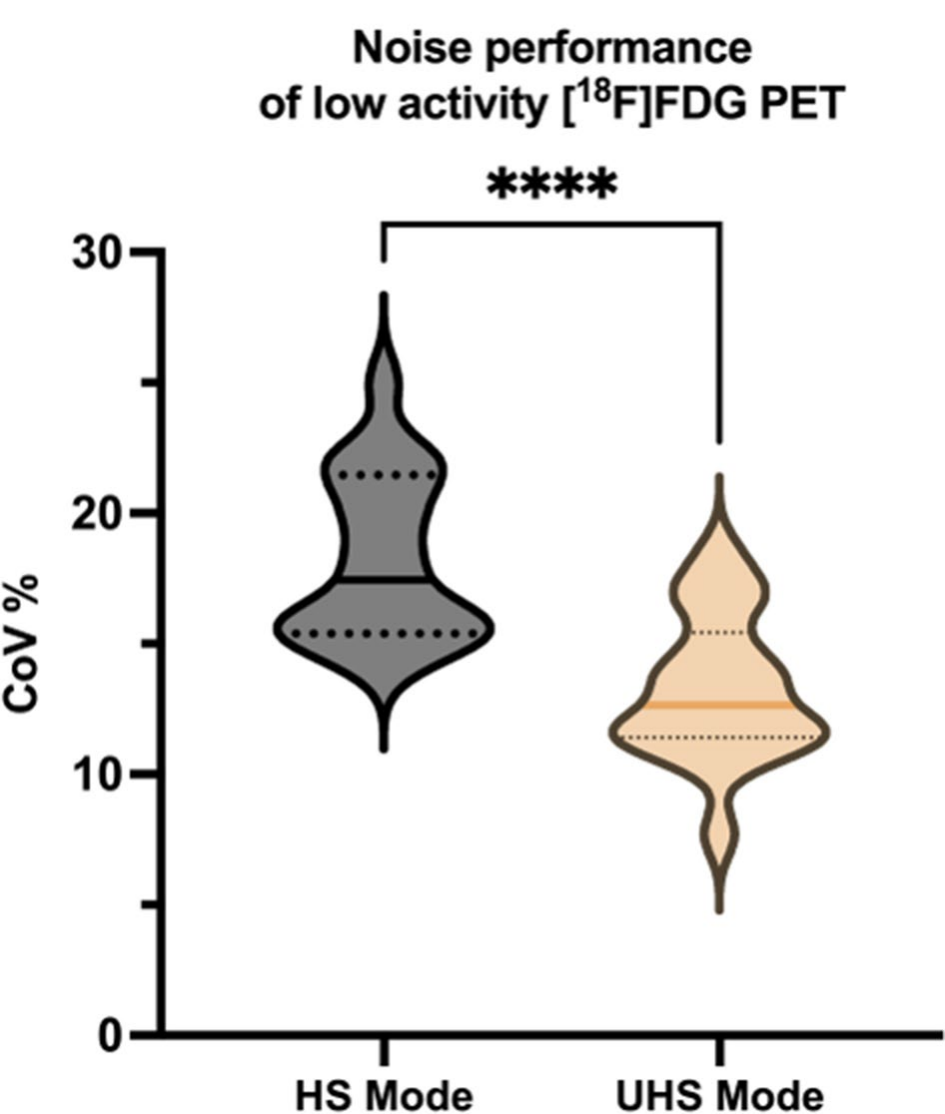
Violin plots displaying the coefficient of variation (CoV) percentage values of the liver background of low activity [^18^F]FDG PET images. Images reconstructed in the high-sensitivity mode (HS) showed a mean CoV of 18.2 ± 3.2%. In comparison, CoV values of images in ultra-high sensitivity mode (UHS) were statistically significantly lower (mean 13.2 ± 2.8%, *p* < 0.001), denoting the improved noise performance of UHS mode. Wilcoxon matched-pairs signed rank test was used to compare variables. The bold line represents the median, the upper dotted line represents the 75th percentile, and the lower dotted line represents the 25th percentile. **** refers to p < 0.001

The mean ACTs of the tumor lesions in the [^18^F]FDG PET scan was 1820 ± 152.7 Bq/mL. The estimated residual [^18^F]FDG activity was 273.3 ± 22.9 Bq/mL for R15% and 364.4 ± 30.5 Bq/mL for R20%. In contrast, the measured mean ACTs of all tumor lesions in the [^18^F]SiFA*lin*-TATE PET scan were significantly higher (13882 ± 1073 Bq/; *p* < 0.0001). This data indicates that residual [^18^F]FDG ACTs resulted in only a minor proportion of activity concentrations of tumor lesions in the [^18^F]SiFA*lin-*TATE PET at 5.2 ± 0.9% and 6.8 ± 1.2% for R15% and R20%, respectively. R15% and R20% exert only a minimal influence on the semi-quantification of [^18^F]SiFA*lin*-TATE. Consequently, there would be no relevant clinical implications, such as incorrect classification of tumor lesions. Furthermore, the mean CoV in the healthy liver tissue in the low activity [^18^F]FDG PET images in HS and UHS was 18.2 ± 0.7% and 13.3 ± 0.6%, respectively. CoV values in UHS were statistically significantly lower compared to HS values, denoting the improved noise performance of UHS mode (*p* < 0.001), which clearly supports low activity imaging and complies with the recommended values by the EANM/EFOMP (Fig. 2).

**Fig. 3:**
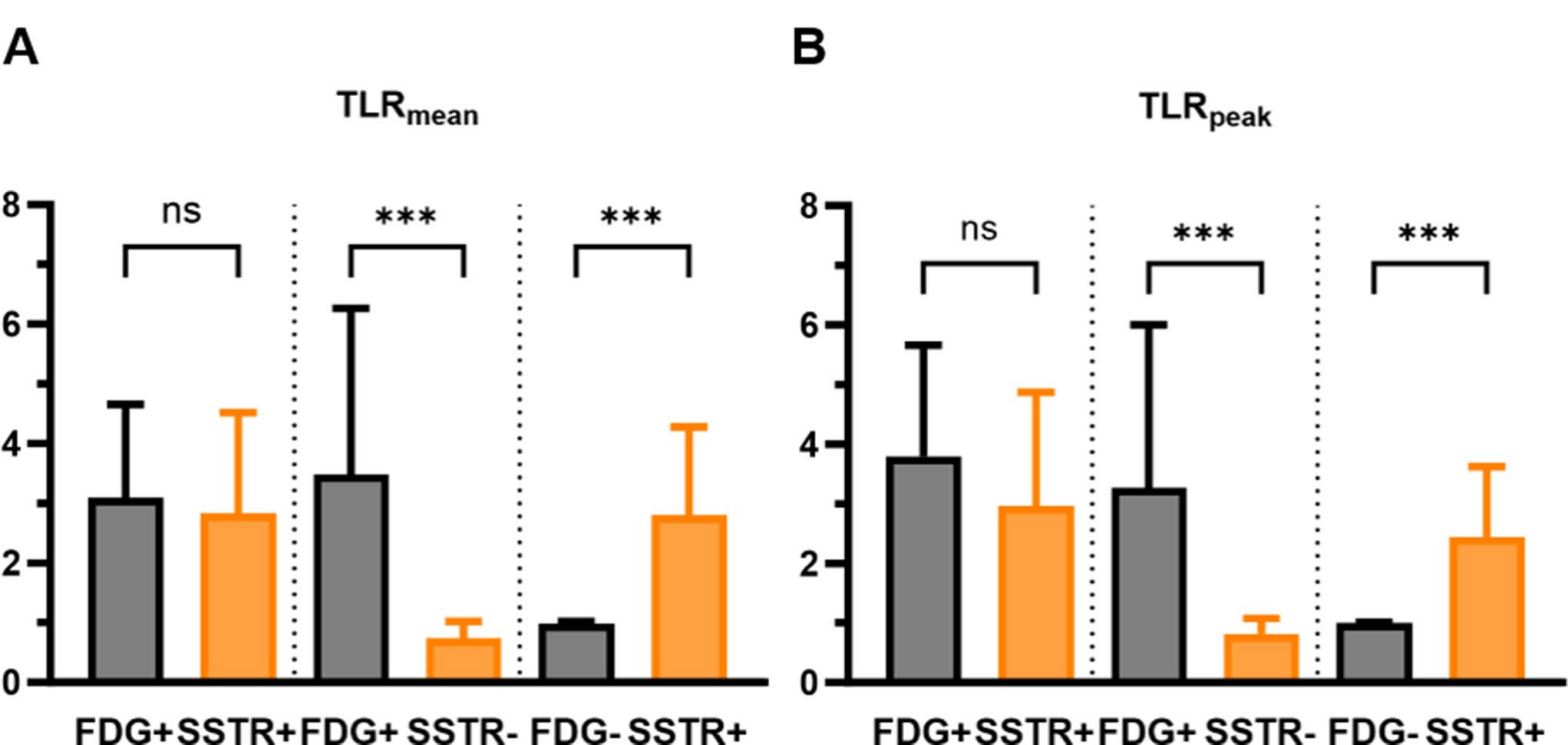
TLR_mean_ (**A**) and TLR_peak_ (**B**) results of all 125 analyzed lesions. Regarding their [^18^F]FDG and [^18^F]SiFA*lin*-TATE uptake, lesions were classified as FDG+/SSTR-, FDG+/SSTR+, and FDG-/SSTR+. Each bar represents the mean, while the error bars represent the standard deviation. The Kruskal-Wallis test was used to compare the difference in median uptake values between lesions in each classification. TLR = tumor-to-liver ratio. ns = non-significant. *** refers to p < 0.001

### Tumor lesion classification and semi-quantification

Four patients showed no tumor lesions on either PET scan after surgical resection of known tumor/metastases, chemotherapy, selective internal radiation therapy (SIRT), or PRRT. Thirteen patients (65%) had evidence of [^18^F]FDG-avid tumor lesions, highlighting the high proportion of [^18^F]FDG-avid disease burden in our cohort of NEN patients. Furthermore, nine patients (45%) had evidence of tumor lesions with increased [^18^F]FDG uptake and no relevant SSTR expression (FDG+/SSTR-), nine patients (45%) showed lesions with increased SSTR expression, but no [^18^F]FDG avidity (FDG-/SSTR+), and seven patients (35%) showed tumor lesions with concordant tracer uptake (FDG+/SSTR+). Overall, one hundred and twenty-five tumor lesions were analyzed in both PET scans. 25/125 (20%) of the NEN-lesions showed concordant increased [^18^F]FDG uptake and SSTR expression with a mean TLR_mean_ of 3.1 ± 0.3 and 2.8 ± 0.3 (FDG+/SSTR+), 55/125 (44%) showed discordant uptake with increased [^18^F]FDG uptake and no relevant SSTR expression with a TLR_mean_ of 3.4 ± 0.3 and 0.7 ± 0.03 (FDG+/SSTR-), and 45/125 lesions (36%) showed only increased SSTR expression (FDG-/SSTR+) with a TLR_mean_ of 0.9 ± 0.03 and 2.8 ± 0.2, respectively (Fig. 3). Lesion classification according to dual-tracer uptake and organ distribution can be found in Table 2.

**Table 2:** Distribution of metastatic tumor lesions according to dual-tracer uptake and involved region

Region	FDG + / SSTR +	FDG + / SSTR -	FDG - / SSTR +
Liver	15	16	25
Lymph nodes	1	23	7
Bone	6	1	12
Lung	3	6	0
Other			
Peritoneum	0	5	0
Myocardium	0	2	0
Ovary	0	1	0
Colon/Small intestine	0	1	1
Total	**25**	**55**	**45**

### Impact on clinical management

**Fig. 4:**
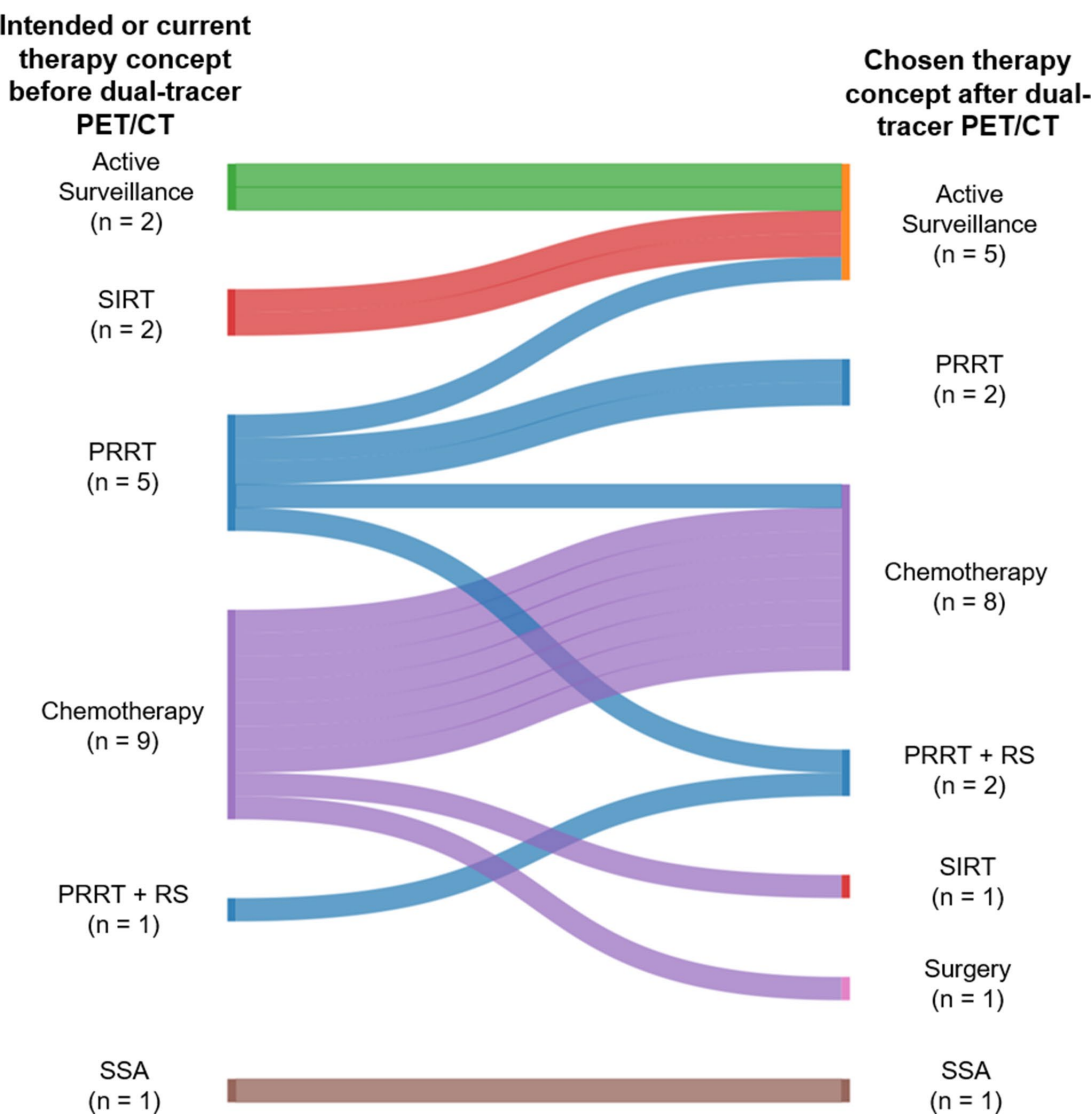
A Sankey diagram illustrating the therapy stratification for patients undergoing dual-tracer PET/CT. 6/20 patients were initially assigned to PRRT ± RS. The initial treatment stratification was maintained in 3/6 patients while a change to therapy intensification was decided in 2/7 patients (*n* = 1 chemotherapy, 1 PRRT ± RS) and therapy de-escalation to AS in one patient. 2/9 patients in the chemotherapy group received locoregional treatments (*n* = 1 SIRT, *n* = 1 surgery), with the majority remaining in the chemotherapy approach. For both patients in the SIRT group, AS approaches were chosen due to the lack of evidence of active disease, whereas dual-tracer PET/CT scans were performed due to suspicious CT findings. SIRT = selective internal radiation therapy; PRRT = peptide receptor radionuclide therapy; RS = Radiosensitizing; SSA = somatostatin analogue; AS = active surveillance; CT = computed tomography

One of the primary factors affecting the treatment stratification was the tumor burden, as assessed by SSTR-MTV and FDG-MTV. These volumes per patient are presented in Table 3. Prior to the dual-tracer PET, the treatment plans included PRRT (*n* = 5), PRRT + RS (*n* = 1), chemotherapy (*n* = 9), active surveillance (AS) (*n* = 2), SIRT (*n* = 2) and SSA (*n* = 1) (Table 3). A Sankey-flow diagram illustrating the treatment stratification is presented in Fig. 4.

**Table 3:** FDG-MTV and SSTR-MTV for each PET examination and implications of dual-tracer PET in therapy stratification

*P*.	FDG MTV (mL)	SSTR-MTV (mL)	G	Therapy concept before DT-PET	Therapy concept after DT-PET	Considered PET information by ITB
1	0	1,2	G2	PRRT	AS	Low SSTR + MTV
2	0	1090	G2	PRRT	PRRT	No discordant MTV (FDG+/STTR-)
3	0	0	G3	AS	AS	No MTV
4	16	0	G2	SIRT	AS	Low extrahepatic [^18^F]FDG MTV
5	0	0	G3	SIRT	AS	No MTV
6	94,6	148	G3	Chx	Chx	FDG + disease; No discordant MTV (FDG+/STTR-)
7	130	0	G3	Chx	Chx Change	Discordant MTV (FDG+/STTR-)
8	568	31,4	G3	Chx	Chx Change	Predominant Discordant MTV (FDG+/STTR-)
9	0	0	G3	SSA	SSA	No MTV
10	3,8	72,8	G3	PRRT	PRRT	No discordant MTV (FDG+/STTR-); FDG + disease
11	1,3	0	G3	PRRT	Chx	Discordant MTV (FDG+/STTR-)
12	0,5	65,2	G2	PRRT + RS	PRRT + RS	No discordant MTV (FDG+/STTR-); FDG + disease
13	0	0	G3	AS	AS	No MTV
14	556	836	G3	PRRT	PRRT + RS	No discordant MTV (FDG+/STTR-); Extensive FDG + disease
15	212	12,8	G2	Chx	SIRT	Discordant MTV (FDG+/STTR-) liver predominant
16	23,2	0	G3	Chx	Surgery	Discordant MTV (FDG+/STTR-)
17	917	61,8	G3	Chx	Chx Change	Predominant Discordant MTV (FDG+/STTR-)
18	122	0	G3	Chx	Chx	Discordant MTV (FDG+/STTR-)
19	236	0	G3	Chx	Chx Change	Discordant MTV (FDG+/STTR-)
20	0	616	G3	Chx	Chx	No discordant MTV (FDG+/STTR-)

P. = Patient; G = Grading; DT-PET = dual-tracer PET; ITB = interdisciplinary tumor board; PRRT = peptide receptor radionuclide therapy; AS = active surveillance; SIRT = selective internal radiotherapy; Chx = Chemotherapy; SSA = somatostatin analogue

**Fig. 5:**
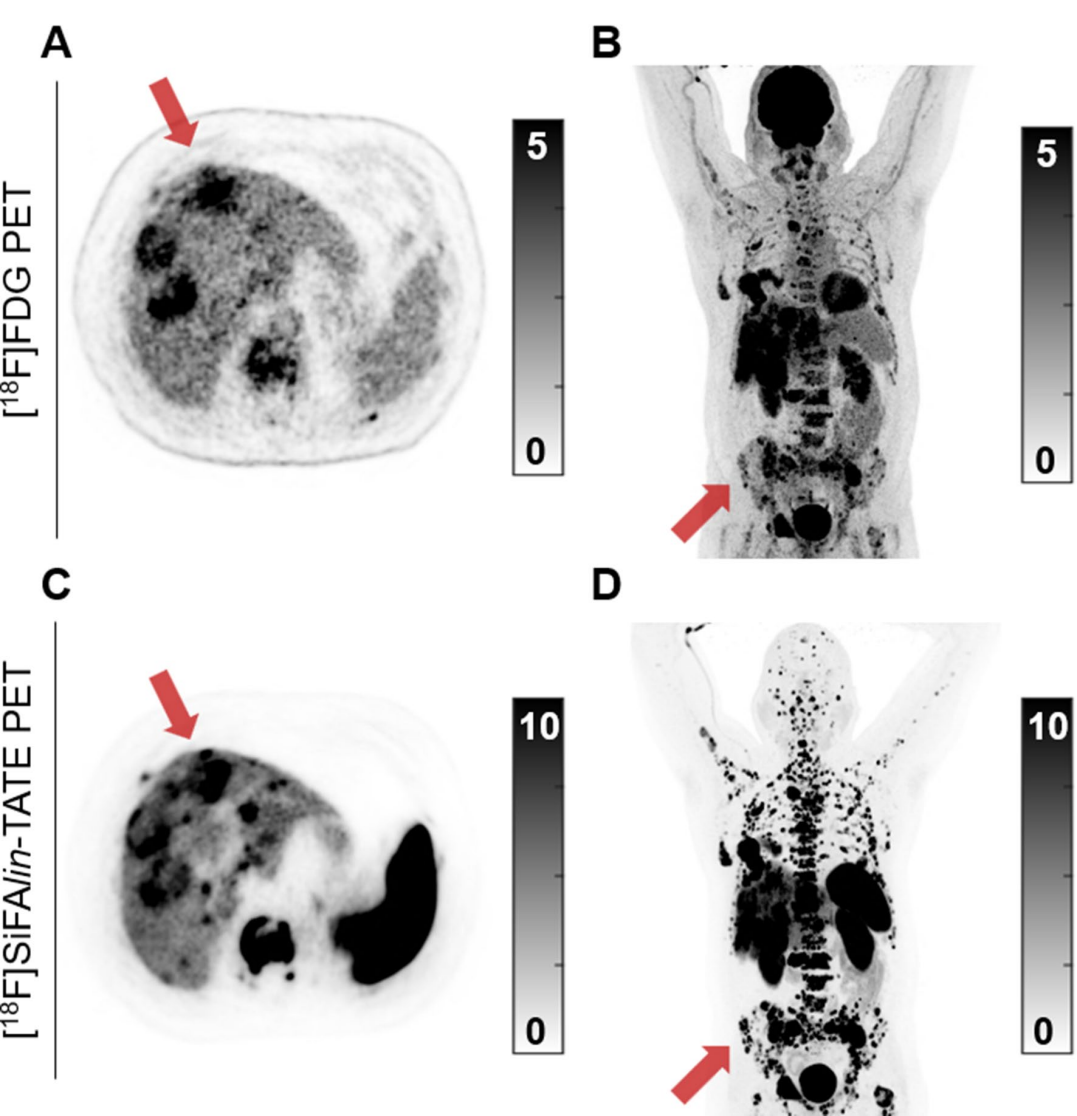
Axial PET images and maximum intensity projections of the low activity [^18^F]FDG PET (69 MBq) (**A**, **B**) and subsequent standard activity [^18^F]SiFA*lin*-TATE PET (367 MBq) (**C**, **D**) of a patient with newly diagnosed CUP NET G2 (Ki67 13.4%). A significant tumor burden is noted in this patient (SSTR-MTV: 836 mL; FDG-MTV: 556 mL), predominantly affecting the liver and bones. Additionally, numerous lesions exhibit concordant uptake of both tracers (FDG+/SSTR+), highlighted by the **red arrows**, which suggests increased tumor aggressiveness and a poorer prognosis. Therefore, a more aggressive treatment plan combining PRRT with RS was recommended and subsequently administered

In the PRRT group (*n* = 6; PRRT and PRRT + RS), dual-tracer PET supported the previously considered treatment in three patients primarily by excluding discordant FDG+/SSTR- lesions (patients 2, 10, 12). In the remaining three patients, dual-tracer PET findings changed their treatment plans. In patient 1, only a low SSTR-MTV and no FDG-MTV were detected, so PRRT was not performed, and AS was initiated. In patient 11, discordant tracer uptake (FDG+/SSTR-) led to a change in treatment strategy to chemotherapy. Finally, in patient 14, a high SSTR + tumor burden with concordant [^18^F]FDG uptake was detected (Fig. 5), prompting the ITB to recommend intensified PRRT combined with RS.

In the chemotherapy group (*n* = 9), dual-tracer PET led to treatment plan changes to locoregional approaches in two patients (*n* = 1 SIRT, *n* = 1 surgery). In patient 15, due to a large discordant tumor volume (FDG+/SSTR-) identified in the liver, SIRT was chosen, while SSAs were introduced to address the low extrahepatic SSTR-MTV. In patient 16, surgical resection of tumor lesions was conducted as an individualized treatment.

**Fig. 6:**
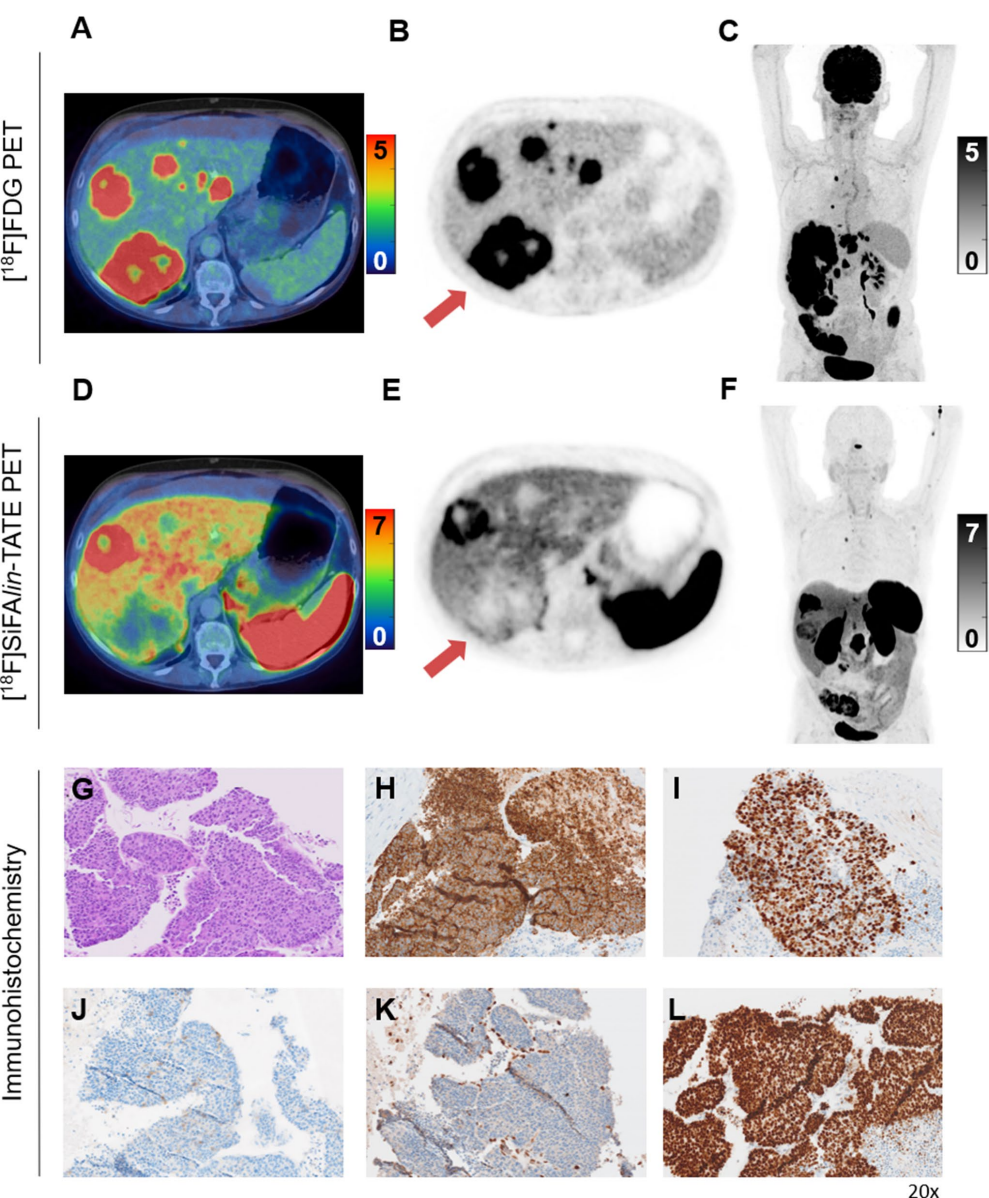
Axial PET images and maximum intensity projections of low activity [^18^F]FDG PET (27 MBq) (**A**-**C**) followed six hours later by a standard activity [^18^F]SiFA*lin*-TATE PET (167 MBq) (**D**-**F**). In this 65-year-old female patient initially diagnosed with a G3 colon NET, a dual-tracer PET/CT was conducted following indications of progressive disease. To further evaluate tumor aggressiveness, a PET-targeted biopsy of a liver lesion exhibiting discordant tracer uptake was carried out (**red arrow**). Histopathological analysis with H&E staining (**G**), positivity for Synaptophysin (**H**), a Ki67 of 49.7% (**I**), negativity for SSTR2 (**J**), and a pathognomonic combination of RB loss (**K**) together with an abnormal p53 expression (**L**), confirmed tumor dedifferentiation into NEC

From the remaining patients (*n* = 7), four patients also benefited from dual-tracer PET due to the delineation of extensive discordant tumor disease (FDG+/SSTR-), receiving regimen adaptations primarily based on disease progression or new histological findings. For example, patient 17, with an initial diagnosis of a colon NET G3 (Ki-67 30%), had been treated with first-line chemotherapy (FOLFOX) according to German NET guidelines [27]. Dual-tracer PET/CT revealed progressive disease with multiple FDG+/SSTR- metastases. Subsequently, a PET-guided biopsy of one FDG+/SSTR- liver metastasis was performed (**red arrow**, Fig. 6E), leading to the histologic diagnosis of NEC (Ki67 49.7%) and initiation of carboplatin-etoposide chemotherapy (Fig. 6G-L). In the SIRT group (*n* = 2), AS was introduced because there was either no evidence of disease or only minimal extrahepatic FDG-MTV. Additionally, patients scheduled for AS and SSAs did not experience any modifications to their treatment plans. In summary, the findings from dual-tracer PET/CT scans directly impacted the therapy stratification of eleven patients (55%).

## Discussion

In cases of high-proliferating NENs, adequate patient management often requires additional [^18^F]FDG PET in conjunction with an SSTR-PET, as outlined in the ESMO clinical practice guidelines [7]. However, this has proven challenging to implement in routine clinical practice. Following the promising results of the recent NETTER2 trial in untreated G2 and G3 NEN patients (Ki67 ≥ 10%), PRRT may now be the treatment of choice for this population, which aligns with the patient cohort presented in this study. An accurate patient stratification will be necessary to better take advantage of this expensive treatment, where combined PET imaging with [^18^F]FDG and radiolabeled SSAs can be of great importance.

Accordingly, the need for timely dual-tracer PET/CT scans for initial staging will increase. Due to the disadvantages of ^68^Ge/^68^Ga-generators involving high costs and low production yields, ^18^F-labeled tracers, such as [^18^F]SiFA*lin*-TATE, are on the rise [6]. Until now, due to the 109.8 min half-life of fluorine-18, the acquisition of two PET scans with ^18^F-labeled radiotracers necessitates a second visit to a nuclear medicine center on a separate day. This would result in an additional burden for cancer patients, who are already under psychological and physical stress. Particularly in this subset of untreated, rapidly progressing NENs, an inappropriate delay in treatment initiation and characterization of tumor biology is a considerable issue. However, by exploiting the increased sensitivity of LAFOV PET/CT scanners, a significant injected activity reduction of the first administered radiotracer may shorten the interval between two separate PET examinations, providing a viable solution [18].

This work proposes a one-day dual-tracer protocol with a low activity [^18^F]FDG and a standard activity [^18^F]SiFA*lin*-TATE PET for patients with high-proliferating NENs. The protocol foresees a 10 min scan with reduced activity of 0.5 MBq/kg [^18^F]FDG PET and a subsequent 5 min scan with 3.0 MBq/kg [^18^F]SiFA*lin*-TATE PET.

Our findings demonstrated that tissue with high physiological glucose metabolism (myocardium and brain) without physiological SSTR expression showed no relevant residual activity in the second PET scan. In the case of the myocardium, the amount of assessable tracer uptake did not differ significantly from that in the blood pool, indicating that measured values correspond to blood pool activity. Furthermore, we analyzed the impact of the residual tumor ACTs of [^18^F]FDG in the second scan. The estimated residual ACT of R20%, which considers the physical decay of fluorine-18 and a potential increase of [^18^F]FDG uptake over time, resulted in an only minor percentual proportion of 6.8 ± 1.2% of the measured ACTs in the [^18^F]SiFA*lin*-TATE PET. Both physiological uptake and tumor ACTs assessments illustrate that the potential residual activity has no significant influence on [^18^F]SiFA*lin*-TATE semi-quantification, even considering this conservative approach. This is an essential aspect of the proposed protocol, as the accurate non-invasive assessment of SSTR expression is paramount for treatment stratification in NENs.

Regarding noise performance and image quality, we recommend using the UHS mode of the Biograph Vision Quadra for the low activity [^18^F]FDG scan. The increased event statistics lead to a reduction in image noise (CoV of 13.3 ± 0.6% (UHS), 18.2% ± 0.7% (HS)), which outweighs the slight degradation of the spatial resolution due to the parallax error [28]. Furthermore, optimizing time intervals between examinations may be feasible, including approaches employing correction methods that account for the physical decay and tracer kinetics to mitigate the impact of tracer “spill-over”.

In our patient cohort, 55% of patients had their management plans altered due to dual-tracer PET findings. This was mainly due to the identification of tumor lesions with discordant tracer uptake, which would not have been assessed by SSTR-PET alone. This demonstrates the meaningful application of dual-tracer approaches in G2/G3 NENs and aligns with the findings of other studies demonstrating the importance of combining [^18^F]FDG and SSTR-PET [13, 29].

Moreover, the assessment of metabolic and molecular tumor volume has shown to be a relevant prognostic factor [30–32], also when specifically evaluating discordant tumor volumes, i.e., consideration of only [^18^F]FDG-avid disease without relevant SSTR expression, which is also related to poorer overall survival in GEP-NENs [24]. Furthermore, as shown in the patient in Fig. 6, dual-tracer PET approaches may guide histologic sampling, allowing the identification of more aggressive tumor sites with increased glucose metabolism that will require more aggressive or multimodal therapeutic regimes. Our streamlined approach can benefit research and clinical studies and improve patient comfort while reducing the logistical challenges of dual-tracer PET examinations. On the patient’s experience side, although a directed evaluation of patient comfort was not performed, on-site patient feedback was generally positive. This is especially relevant since, as an ENETS Center of Excellence, many patients have long travel times to our facility and often have to arrange solutions for related issues, such as companions in the case of sedation requirements due to claustrophobia or arranging childcare. Because of these issues, on-site patient feedback was generally positive since both examinations could be completed in one visit.

The proposed imaging procedure might also be applied to different radiotracers, including using radionuclides other than fluorine-18 or involving other tumor entities, such as prostate cancer, i.e., by combining [^18^F]FDG and [^18^F]PSMA-1007. The recent TheraP study highlighted the potential benefit of combining [^18^F]FDG and PSMA-PET for treatment stratification and prognosis assessment since patients displaying [^18^F]FDG-avid discordant disease generally showed a shorter median overall survival [33]. Regarding prostate cancer patients, a dual-tracer protocol in a single imaging session combining [^68^Ga]Ga-PSMA-11 and [^18^F]FDG has already been developed for the Biograph Vision Quadra by Alberts et al. [34], allowing the detection of mismatch lesions. However, this protocol differs significantly from the current study, as quantification of the second radiotracer is not achievable due to the short time interval between scans and the resulting radiotracer superimposition (low activity [^18^F]FDG PET being performed 1 h after the conclusion of the PSMA-PET).

LAFOV PET/CT scanners are increasingly being installed in different hospitals worldwide because of their significantly increased sensitivity, leading to many clinical advantages. Several studies have shown that reductions of the injected activity are feasible using LAFOV PET scanners, which are undoubtedly worthwhile for pediatric examinations [35, 36]. Still, some critics question whether the reduction in radiation exposure may be negligible in adult oncological patients. However, performing two ^18^F-labeled PET scans in one day could be considered a clinical “killer application” for LAFOV PET/CT scanners, facilitating quantifiable, one-day multi-tracer PET examinations. Furthermore, we deem scalability feasible due to the reduced acquisition times of low activity [^18^F]FDG PET scans and the generally higher patient throughput that can be attained with LAFOV PET/CT scanners. Our study showed that the proposed protocol was feasible, even if the limited number of patients reported in this study must be considered a potential limitation.

## Conclusion

Our study demonstrates that LAFOV PET scanners enable a one-day dual-tracer protocol with good image quality and preserved semi-quantification of two ^18^F-labeled radiotracers. Our approach facilitates the assessment of tumor biology and tumor heterogeneity while reducing the logistical burden. In addition, we claim that one real advantage of low activity PET imaging is enabling one-day dual-tracer PET examinations.

## Electronic supplementary material

Below is the link to the electronic supplementary material.

Supplementary Material 1

## Data Availability

The datasets generated during and/or analyzed during the current study are available from the corresponding author on reasonable request.
