# Supplementary material for: One-day dual-tracer examination in neuroendocrine neoplasms: a real advantage of low activity LAFOV PET imaging
Source: Eur J Nucl Med Mol Imaging. 2025 Jan 30;52(7):2463–76. doi: 10.1007/s00259-025-07073-w (PMC12119714; doi:10.1007/s00259-025-07073-w)
Supplement: Supplementary file 1 — Supplementary Material 1 [file 259_2025_7073_MOESM1_ESM.docx]

**Article Title:** One-day dual-tracer examination in neuroendocrine neoplasms: a real advantage of low activity LAFOV PET imaging.

**Journal:** European Journal of Nuclear Medicine and Molecular Imaging.

Eduardo Calderón^1^, Lena S. Kiefer^1^, Fabian P. Schmidt^1,2^, Wenhong Lan^1^, Andreas S. Brendlin^3^, Christian P. Reinert^3^, Stephan Singer^4^, Gerald Reischl^2,7^, Martina Hinterleitner^5,6,7^, Helmut Dittmann^1,6^, Christian la Fougère^1,6,7,8^, Nils F. Trautwein^1,6^

^1^ University Hospital Tuebingen, Department of Nuclear Medicine and Clinical Molecular Imaging, Otfried-Mueller-Str. 14, 72076, Tuebingen, Germany.

^2^ Werner Siemens Imaging Center, Department of Preclinical Imaging and Radiopharmacy, Eberhard-Karls University Tuebingen, Röntgenweg 13, 72076, Tuebingen, Germany.

^3^ University Hospital Tuebingen, Department of Diagnostic and Interventional Radiology, Hoppe-Seyler-Str. 3, 72076, Tuebingen, Germany.

^4^ University Hospital Tuebingen, Department of Pathology, Liebermeisterstr. 8, 72076, Tuebingen, Germany.

^5^ University Hospital Tuebingen, Department of Medical Oncology and Pneumology (Internal Medicine VIII), Otfried-Mueller-Str. 14, 72076, Tuebingen, Germany

^6^ University Hospital Tuebingen, ENETS Center of Excellence, Otfried-Mueller-Str. 14, 72076, Tuebingen, Germany

^7^ DFG Cluster of Excellence 2180 ‘Image-Guided and Functional Instructed Tumor Therapy’ (iFIT), University of Tuebingen, Roentgenweg 11, 72076, Tuebingen, Germany

^8^ German Cancer Consortium (DKTK), German Cancer Research Center (DKFZ) Partner Site Tuebingen, Auf der Morgenstelle 15, 72076, Tuebingen, Germany

| Address for Correspondence: |
| --- |
| Christian la Fougère  Department of Nuclear Medicine and Clinical Molecular Imaging  Otfried-Müller-Straße 14, 72076 Tübingen  Phone: +49 07071 29-86553  Fax: +49 07071 29-4601  Email: christian.lafougere@med.uni-tuebingen.de  ORCID: https://orcid.org/0000-0001-7519-0417 |

**SUPPLEMENTARY MATERIAL**

**Supplementary Table 1:**Specifications regarding imaging procedure and elapsed time between PET scans of all patients.

| Patient | Bodyweight  (kg) | Blood glucose  (mg/dl) | Injected [^18^F]FDG Dose (MBq) | Injected [^18^F]FDG per bodyweight (MBq/kg) | [^18^F]FDG uptake time (Minutes) | Time to 2^nd^ Tracer injection including uptake time (Minutes) | Injected [^18^F]SiFA*lin*-TATE Dose (MBq) | Injected [^18^F]SiFA*lin*-TATE per bodyweight (MBq/kg) | [^18^F]SiFA*lin*-TATE uptake time  (Minutes) | Total elapsed time between PET scans  (Minutes) | Elapsed half-lives of F-18 between PET scans |
| --- | --- | --- | --- | --- | --- | --- | --- | --- | --- | --- | --- |
| 1 | 63 | 96 | 38 | 0.6 | 60 | 296 | 179 | 2.9 | 90 | 326 | 3.0 |
| 2 | 80 | 87 | 48 | 0.6 | 60 | 347 | 262 | 3.3 | 90 | 377 | 3.4 |
| 3 | 54 | 75 | 25 | 0.5 | 60 | 268 | 166 | 3.1 | 90 | 298 | 2.7 |
| 4 | 74 | 102 | 33 | 0.4 | 60 | 301 | 249 | 3.3 | 90 | 331 | 3.0 |
| 5 | 62 | 104 | 29 | 0.5 | 60 | 316 | 191 | 3.1 | 90 | 346 | 3.2 |
| 6 | 48 | 96 | 25 | 0.5 | 60 | 326 | 138 | 2.9 | 90 | 356 | 3,2 |
| 7 | 93 | 93 | 53 | 0.6 | 60 | 303 | 261 | 2.8 | 90 | 333 | 3.0 |
| 8 | 77 | 75 | 33 | 0.4 | 60 | 304 | 214 | 2.8 | 90 | 334 | 3.0 |
| 9 | 73 | 145 | 45 | 0.6 | 60 | 280 | 232 | 3.2 | 90 | 310 | 2.8 |
| 10 | 44 | 99 | 25 | 0.6 | 60 | 347 | 153 | 3.5 | 90 | 377 | 3.4 |
| 11 | 53 | 94 | 30 | 0.6 | 60 | 310 | 169 | 3.2 | 90 | 340 | 3.1 |
| 12 | 80 | 177 | 45 | 0.6 | 60 | 308 | 241 | 3.0 | 90 | 338 | 3.1 |
| 13 | 83 | 112 | 47 | 0.6 | 60 | 289 | 244 | 3.0 | 90 | 319 | 2.9 |
| 14 | 122 | 90 | 69 | 0.6 | 60 | 295 | 367 | 3.0 | 90 | 325 | 3.0 |
| 15 | 110 | 80 | 52 | 0.5 | 60 | 280 | 340 | 3.1 | 90 | 310 | 2.8 |
| 16 | 60 | 110 | 33 | 0.6 | 60 | 330 | 176 | 2.9 | 90 | 360 | 3.3 |
| 17 | 50 | 102 | 27 | 0.5 | 60 | 311 | 167 | 3.3 | 90 | 341 | 3.1 |
| 18 | 90 | 103 | 47 | 0.5 | 60 | 361 | 292 | 3.2 | 90 | 391 | 3.6 |
| 19 | 83 | 122 | 45 | 0.5 | 60 | 285 | 240 | 2.9 | 90 | 315 | 2.9 |
| 20 | 82 | 113 | 41 | 0.5 | 60 | 351 | 160 | 2.0 | 90 | 381 | 3.5 |

**Supplementary Table 2**: Details of the CT imaging protocols.

| Scan parameters diagnostic CT | |
| --- | --- |
| slice thickness | 1 mm |
| pitch factor | 0.3 |
| twin beam dual energy  tube voltage  tube current reference | AuSn120 kV  Ref. mAs (using CARE Dose 4D) |
| contrast media | |
| portal venous phase (90 seconds p.i.) | 80-140 mL Ultravist370 (Schering AG, Berlin, Germany) * |
| native | any contraindications for contrast media |
| Scan parameters low-dose CT |  |
| tube voltage | Sn140 kV |
| tube current | 6 mAs |

*Weight-adapted intravenous injection at 1.0-2.5 mL/s, followed by a 40 mL saline flush
